# Does aphid salivation affect phloem sieve element occlusion *in vivo*?

**DOI:** 10.1093/jxb/ert325

**Published:** 2013-10-14

**Authors:** Karla J. Medina-Ortega, G. P. Walker

**Affiliations:** Department of Entomology, University of California, Riverside, CA 92521, USA

**Keywords:** Electrical penetration graph, EPG, feeding behaviour, forisomes, insect–plant interactions, phloem sealing, plant defence response.

## Abstract

To protect against loss of photo-assimilate-rich phloem sap, plants have evolved several mechanisms to plug phloem sieve tubes in response to damage. In many Fabaceae, each sieve element contains a discrete proteinaceous body called a forisome, which, in response to damage, rapidly transforms from a condensed configuration that does not impede the flow of sap to a dispersed configuration that plugs the sieve element. Aphids and other specialized phloem sap feeders can ingest phloem sap from a single sieve element for hours or days, and to do this, they must be able to suppress or reverse phloem plugging. A recent study provided *in vitro* evidence that aphid saliva can reverse forisome plugs. The present study tested this hypothesis *in vivo* by inducing forisome plugs which triggered aphids to switch behaviour from phloem sap ingestion to salivation into the sieve element. After salivating into the sieve element for various periods of time, the aphids were instantaneously cryofixed (freeze fixed) *in situ* on their leaf. The state of the forisome was then determined in the penetrated sieve element and in nearby non-penetrated sieve elements which served as controls for sieve elements not subjected to direct aphid salivation. Forisomes were almost always in close contact with the stylet tips and thus came into direct contact with the saliva. Nonetheless, forisome plugs in the penetrated sieve element did not revert back to a non-plugging state any faster than those in neighbouring sieve elements that were not subjected to direct aphid salivation.

## Introduction

In plants, phloem is the transport system for mobilization of photo-assimilates from sources to sinks. Several cell types comprise the phloem, and phloem sap is transported mainly in sieve elements, which are specialized elongate cells that abut end-to-end to form continuous tubes (sieve tubes). Cell walls between adjacent sieve elements in a sieve tube are perforated with a high density of modified plasmodesmata, called sieve pores, and are referred to as sieve plates (Parthasarathy, 1975; Cronshaw, 1981; Schulz, 1998). Sieve plates provide a high degree of cytoplasmic connections between adjacent sieve elements, allowing a continuous flow of sap through sieve tubes. Mass flow of sap through sieve tubes is driven by a differential pressure between sources and sinks (Münch, 1930; Goeschl and Magnuson, 1986; Magnuson *et al.*, 1986).

Plants have two well-known phloem occlusion mechanisms to prevent loss of sap upon disturbance or damage to the phloem: callose deposition in sieve pores and formation of phloem protein (P-protein) plugs that accumulate on the sieve plates (Eschrich, 1975; van Bel, 2006). Callose deposition requires *de-novo* synthesis and takes several minutes to build up enough deposition to occlude the sieve pores (Furch *et al.*, 2007). Unlike callose, P-proteins do not need to be synthesized upon damage as they are constitutively present in mature sieve elements (Cronshaw, 1981) and can form plugs in less than a second after damage (Knoblauch and van Bel, 1998; Knoblauch *et al.*, 2001; Knoblauch and Peters, 2004). Sieve element occlusion by callose and P-protein can be reversible (Knoblauch *et al.*, 2001; Furch *et al.*, 2007, 2009), so that if the disturbance does not kill the sieve element, the sieve element can resume transportation of sap. Callose deposition in the sieve pores can be degraded in about 30min (Furch *et al.*, 2007), and dissolution of P-protein plugs can be as fast as 3–8min (data herein).

P-protein is a collective term for phloem-specific structural proteins that occur in sieve elements, and P-proteins have been detected in all dicotyledons studied to date (Cronshaw and Sabnis, 1990). More recently, the term ‘sieve element occlusion (SEO) protein’ has been proposed for P-proteins that plug sieve elements in response to damage (Pélissier *et al.*, 2008). So far, there are no known P-proteins other than SEO proteins. In many Fabaceae, SEO proteins occur as discrete spindled shape bodies originally referred to as crystalline P-protein bodies (Cronshaw and Sabnis, 1990) and more recently have been renamed as forisomes (Knoblauch *et al.*, 2003). In translocating sieve elements, forisomes are in a condensed state that does not interfere with the flow of sap, but in response to perturbation to the sieve element, they instantaneously switch to a dispersed state that plugs the sieve element and interferes with the flow of sap (Knoblauch *et al.*, 2001; Knoblauch and Peters, 2004). The SEO protein plugging mechanism is very sensitive, and perturbation in one sieve element can cause SEO protein occlusion in neighbouring sieve elements (Knoblauch and van Bel, 1998).

Aphids are one of the most damaging groups of agricultural pests. They damage plants directly by the effects of their saliva on the plant and by extracting phloem sap; they also damage plants indirectly by transmission of many plant viruses. Aphid mouthparts consist of piercing stylets that penetrate the plant and navigate intercellularly to reach phloem sieve elements from which they ingest sap (Tjallingii and Hogen Esch, 1993). Their success as specialized phloem sap feeders is due to their ability to pierce sieve elements and ingest phloem sap uninterrupted for hours or even days (Tjallingii, 1995; Douglas, 2006). To accomplish this feat, they must be able to sustain sap flow through the sieve elements without triggering phloem plugging and/or be able to reverse phloem plugging. Aphids always inject saliva into a sieve element immediately after penetrating it (Prado and Tjallingii, 1994). Consequently, it has been hypothesized that salivation after penetrating a sieve element functions to reverse, suppress, or prevent the SEO protein plug that would otherwise be induced by penetration (Will and van Bel, 2006; Will *et al.*, 2009). However, Walker and Medina-Ortega (2012) recently demonstrated that at least for pea aphid (*Acyrthosiphon pisum* [Harris]), feeding on faba bean (*Vicia faba* L.), penetration of a sieve element does not trigger formation of SEO protein plugs.

To study the effects of sieve element occlusion on aphid feeding and vice versa, sieve element occlusion can be experimentally induced (Furch *et al.*, 2007, 2009). Application of a burn stimulus to the terminal end of a vein initiates an electrical potential wave which travels down the length of the vein, causing an influx of Ca^2+^ into the sieve elements, which in turn triggers forisome dispersal (Furch *et al.*, 2007; Hafke and van Bel, 2013). This is a very useful technique for studying interactions between sieve element occlusion and aphid feeding because an aphid feeding several centimetres away from the vein apex, where the burn is applied, will be affected by the electrical potential wave and forisome dispersal, not by the burn itself. Will *et al.* (2007) used this technique while recording electrical penetration graphs (EPGs) from aphids (*Megoura viciae* Buckton) feeding on faba bean midrib veins and found that aphids switched from phloem ingestion (EPG waveform E2) to phloem salivation (EPG waveform E1) several seconds after application of a burn stimulus at the tip of the midrib. After about 8min of salivation, the aphids resumed phloem ingestion. They hypothesized that the aphids switched from ingestion to salivation in response to forisome dispersal triggered by the remote burn stimulus and that they eventually resumed ingestion because the saliva reversed the forisome plugs. However, they did not examine the state of the forisomes to verify this hypothesis. They supported their hypothesis by concentrating saliva collected from artificial diet fed upon by thousands of *M. viciae* and applying the concentrated saliva to isolated *V. faba* forisomes *in vitro*. When the concentrated saliva was applied to forisomes in a dispersed (plugging) state, the forisomes condensed to a non-plugging state.

The study presented here tested the hypothesis that aphid salivation reverses remote burn-induced forisome plugs *in vivo* under the natural condition of a single aphid salivating into a sieve element where the forisome is in a plugged state. Since forisome dispersal is reversible, it was first necessary to determine how long after a remote burn stimulus do forisomes remain in a dispersed plugging state in leaves without any exposure to aphids. The EPG technique was then used to measure how long aphids salivate into sieve elements after forisome plugs are triggered by application of a remote burn stimulus and to determine whether the aphids resumed ingestion or withdrew their stylets from the sieve element after the salivation period. Finally, while aphids were ingesting phloem sap, a remote burn stimulus was applied to trigger forisome dispersal and aphid salivation. Aphids were allowed to salivate into the sieve element for various periods of time, and then the aphid and phloem were instantaneously cryofixed and processed by the techniques of Walker and Medina-Ortega (2012) to determine if the forisome in the penetrated sieve element reverted to a non-plugging state any sooner than forisomes in nearby non-penetrated sieve elements which served as controls where no saliva was injected.

## Materials and methods

### Plant and insect material

Faba bean plants (*V. faba* L. cv. Windsor) were grown in plastic pots containing soil supplemented with Osmocote 14:14:14 fertilizer (Scotts Company, Marysville, OH, USA) in a greenhouse under natural light conditions. The pea aphid, *A. pisum* (Harris), colony was started from a single aphid collected from alfalfa in Stillwater, OK, USA (provided by Dr Jack Dillwith, Oklahoma State University) and was maintained on faba bean (cv. Windsor) in a greenhouse. Experiments usually used pre-flowering plants and apterous adult and third to fourth instar aphids.

### Electrical penetration graph recording

Aphid feeding behaviour was monitored with a Giga-4 or Giga-8 DC-EPG (EPG Systems, Wageningen, The Netherlands) set at ×100 amplification. Recordings were made on intact leaves of potted plants and the substrate electrode was placed in the moist soil. Output signals from the EPG were digitized at a sample rate of 100 Hz per channel with a Dataq 720 analogue to digital converter and recorded with Windaq Pro software (A-D converter and software from Dataq Instruments, Akron, OH, USA). During recording, the substrate voltage was adjusted so that the waveforms fit within a ±5V window provided by the recording software. The whole set up was placed in a Faraday cage to reduce electrical noise.

As described in Walker and Medina-Ortega (2012), a 12.7-μm-diameter gold wire was attached to the dorsum of each aphid with water-soluble silver glue while the aphid was held down by a vacuum device. After aphids were wired, they were placed on midribs or lateral veins on the abaxial side of young fully developed leaves approximately 3cm away from the vein’s terminal end. The abaxial side of the leaf initially faced up when the aphids were placed on the leaf, and after they secured a grip, the leaf was turned over so that during EPG recording, the leaf was in its normal orientation, abaxial side down. Details on how the aphids were confined to the vein of interest and how the orientation of the leaves was manipulated are given in Walker and Medina-Ortega (2012).

### Cryofixation and sample processing

To instantaneously fix the phloem, a cryofixative was poured onto a selected site on the adaxial side of the leaf. For samples where aphids were feeding, the selected site was directly opposite to where the aphid was feeding on the abaxial side. For samples of leaf tissue without aphids and for samples of leaf tissue with aphids feeding on lateral veins, the cryofixative was 95% ethanol chilled to approximately –120 °C with liquid nitrogen as in Walker and Medina-Ortega (2012). This worked well for cryofixing the leaf tissue and for freezing the aphid in place on lateral veins. However, midribs are much thicker than lateral veins and the –120 °C ethanol frequently did not freeze the aphid on the opposite side of the leaf rapidly enough to keep it from pulling out its stylets and falling off the leaf. Consequently for fixing aphids *in situ* on midribs, a much colder cryofixative was used: liquid nitrogen that was placed under a vacuum to lower its temperature beyond its atmospheric pressure boiling point (Froelich *et al.*, 2011).

Following cryofixation, samples were subjected to freeze substitution in 95% ethanol at –78.5 °C for 2 d, and then at –20 °C for at least 1 d. The samples then were removed from the freezer and placed in a Styrofoam container where they slowly warmed to room temperature. Following freeze substitution, samples were dissected and stained for confocal laser-scanning microscopy (CLSM) as described previously (Walker and Medina-Ortega, 2012). Samples were examined using either a Zeiss LSM 510 or a Leica SP 5 CLSM microscope with default settings for FITC/rhodamine double-labelling in the Zeiss or FITC/Texas Red double-labelling in the Leica. Micrographs were taken with a water immersion lens of ×63 and ×40 in the Zeiss and Leica microscopes, respectively.

### Experiment 1: Duration of forisome dispersal after a remote burn stimulus

Experiment 1 determined how long forisomes remain in a dispersed state following a remote burn stimulus when they are not subjected to aphid salivation. Forisome dispersal was triggered in midribs of young, uninfested, fully developed leaves attached to the plant, by burning the leaf tip with a match for about 3 s (Furch *et al.*, 2007), and was triggered in lateral veins by touching the apex of the vein with the tip of a soldering iron for about 3 s. A selected target area on the vein, 3cm basipetally from the burn site, was cryofixed at various times following the remote burn stimulus: 2, 4, 6, 8, or 10min for midribs (2 replicates for each time interval); and <30 s, 1, 2, 2.5, 3, 3.5, 4, or 8min for lateral veins (3–7 replicates per time interval). Each replicate used a leaf from a different plant. After cryofixation, the samples were freeze substituted, dissected, and stained for CLSM as described by Walker and Medina-Ortega (2012). The state of the forisomes (dispersed or condensed) was then determined. The proportion of forisomes in a dispersed state was calculated based on the total number of forisomes observed in each replicate (*n*=11–36 forisomes per replicate).

### Experiment 2: Aphid response to a remote burn stimulus

Aphid feeding behaviour was monitored by EPG on both midribs and lateral veins of leaves intact on the plant, and after the aphid engaged in sustained phloem ingestion (EPG waveform E2) for more than 10min, a remote burn stimulus was applied to the apex of the vein on which the aphid was feeding. The aphid was approximately 3cm basipetally from the burn site. The burn was applied the same way as described in Experiment 1 and always triggered a change in aphid behaviour from phloem sap ingestion to salivation into the sieve element (EPG waveform E1). EPGs were recorded for at least 2h after the burn was applied. The time from application of the burn to the onset of waveform E1 was recorded as well as the duration of waveform E1 following the burn. E1 duration included the time that the aphids were in ‘pure E1’ and the E1/E2 mixture that typically occurs in a transition from E1 to E2 (Will *et al.*, 2007). Waveform E1 eventually ended and was followed by either a return to ingestion (E2) or withdrawal from the sieve element and engagement in pathway phase behaviour (Pettersson *et al.* 2007); the proportion of aphids that returned to E2 and the proportion of those that followed E1 with pathway were recorded. EPG recordings were obtained from 12 aphids feeding on midribs and from 14 aphids feeding on lateral veins. Each replicate used a different aphid and different plant.

The following statistical analyses were conducted using JMP software (version 10.0, SAS Institute). Time from the remote burn to initiation of waveform E1 and duration of E1 were compared between midribs and lateral veins using the nonparametric Wilcoxon test. The proportion of aphids that immediately followed E1 with E2 (as opposed to pathway phase) was compared between midribs and lateral veins using Fisher’s exact test. The duration of E1 for aphids that followed E1 with E2 versus those that followed E1 with pathway was compared using a t-test for unequal variances. The latter test was done only for data from lateral veins because the number of aphids on midribs that followed E1 with E2 was too low to provide a meaningful analysis. These data were square root transformed, which made the residuals normal but still did not eliminate heterogeneity of the variances (tested with the F statistic); hence the t-test for unequal variances.

### Experiment 3: Effect of aphid salivation on dispersed forisome recovery

EPG was used to monitor aphids feeding on the abaxial side of young fully developed leaves intact on the plant, approximately 3cm away from the apex of midribs or lateral veins. After the aphid was ingesting sap from a sieve element for at least 10min (waveform E2), a remote burn was applied to the distal end of the vein as described previously (or for nine samples on lateral veins, the remote burn was applied with the tip of a flame-heated steel probe, 1-mm tip diameter). At various time intervals after the burn, during which the aphids were salivating into the sieve element, the aphid and phloem were cryofixed as described previously. Samples were processed for examination by CLSM as described previously, and the state of the forisome (dispersed, condensed, or intermediate) in the aphid-penetrated sieve element was compared with forisomes in nearby non-penetrated sieve elements. The forisomes in nearby non-penetrated sieve elements served as controls to determine if the forisome subjected to direct aphid salivation recovered any faster than forisomes in nearby non-penetrated sieve elements.

In experiment 1, it was observed that forisomes generally did not disperse and/or recover in response to a remote burn stimulus uniformly across an abaxial–adaxial gradient; consequently, the most appropriate non-penetrated sieve elements for comparison to the penetrated sieve element would be those at the same level in the abaxial–adaxial gradient. Therefore, the ‘nearby’ non-penetrated sieve elements examined were those that could be seen on the confocal microscope by focusing from slightly above to slightly below the forisome in the penetrated sieve element (mean±SD distance 7.7±1.9 μm) in a square field of view (146×146 μm; the field of view provided by the ×63 objective at zoom=1 on the Zeiss LSM 510 confocal microscope) with the forisome in the penetrated sieve element near the centre of the field of view. All forisomes seen in this search area were scored as dispersed, intermediate, or condensed. The number of forisomes in non-penetrated sieve elements found within the search area varied among samples; consequently, the proportion of forisomes in each state for the non-penetrated sieve elements was calculated two ways: (1) the total number of forisomes in each state was summed over all the samples and then the proportions were calculated from these sums; or (2) the proportions of forisomes in each state were calculated for each sample and then these proportions were used to calculate an average over all samples.

## Results

### Experiment 1: Duration of forisome dispersal after a remote burn stimulus

**Fig. 1.**
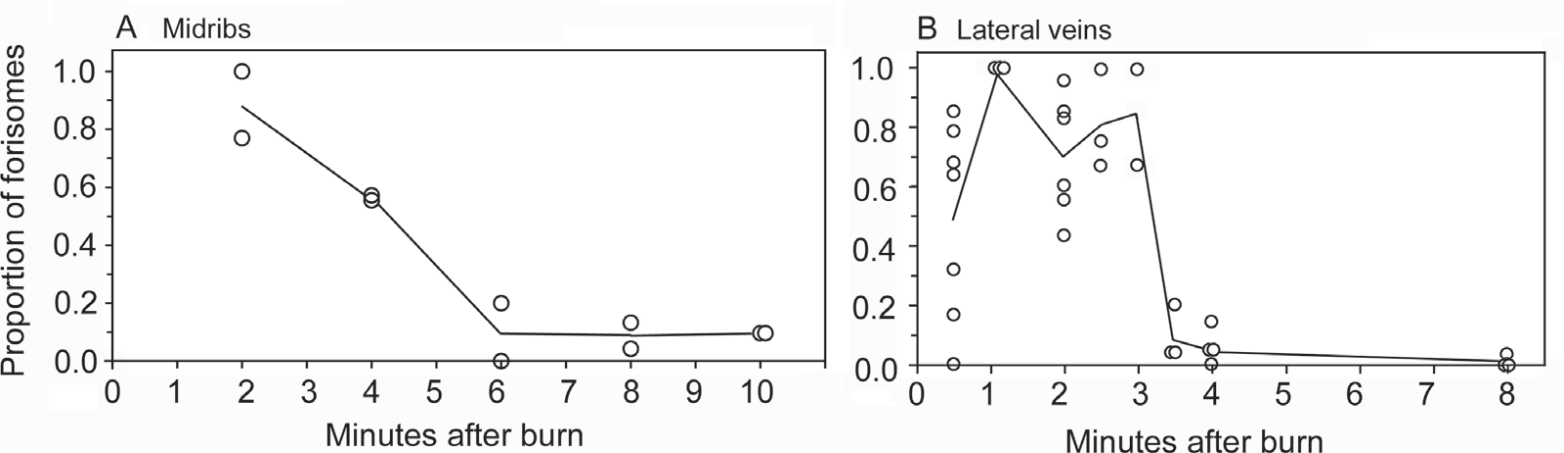
Proportion of forisomes in a dispersed state in midribs (A) and lateral veins (B) at different times after application of a remote burn stimulus. Circles plot data from individual replicates (*n*=11–36 forisomes examined in each replicate). Lines plot average of all replicates at each time. In controls (no remote burn), proportion of forisomes in a dispersed state was zero in all replicates for both vein types (*n*=2 replicates for midribs and *n*=3 replicates for lateral veins; data not plotted).

In Figure 1, the proportion of forisomes that were in a dispersed state is plotted as a function of time after application of a remote burn stimulus. The data indicate that forisomes recovered faster on lateral veins than on midribs. On lateral veins, the proportion of forisomes that were dispersed dropped down sharply to about 0.10 between 3 and 3.5min (Fig. 1). In contrast on midribs, the proportion of forisomes that were dispersed was still greater than 0.50 by 4min after the remote burn stimulus. By 6min after burning on midribs, the proportion of forisomes that were dispersed was down to about 0.10. The proportion of forisomes in a dispersed state in the controls (no remote burn) was zero for all replicates for both vein types.

### Experiment 2: Aphid response to a remote burn stimulus

**Fig. 2.**
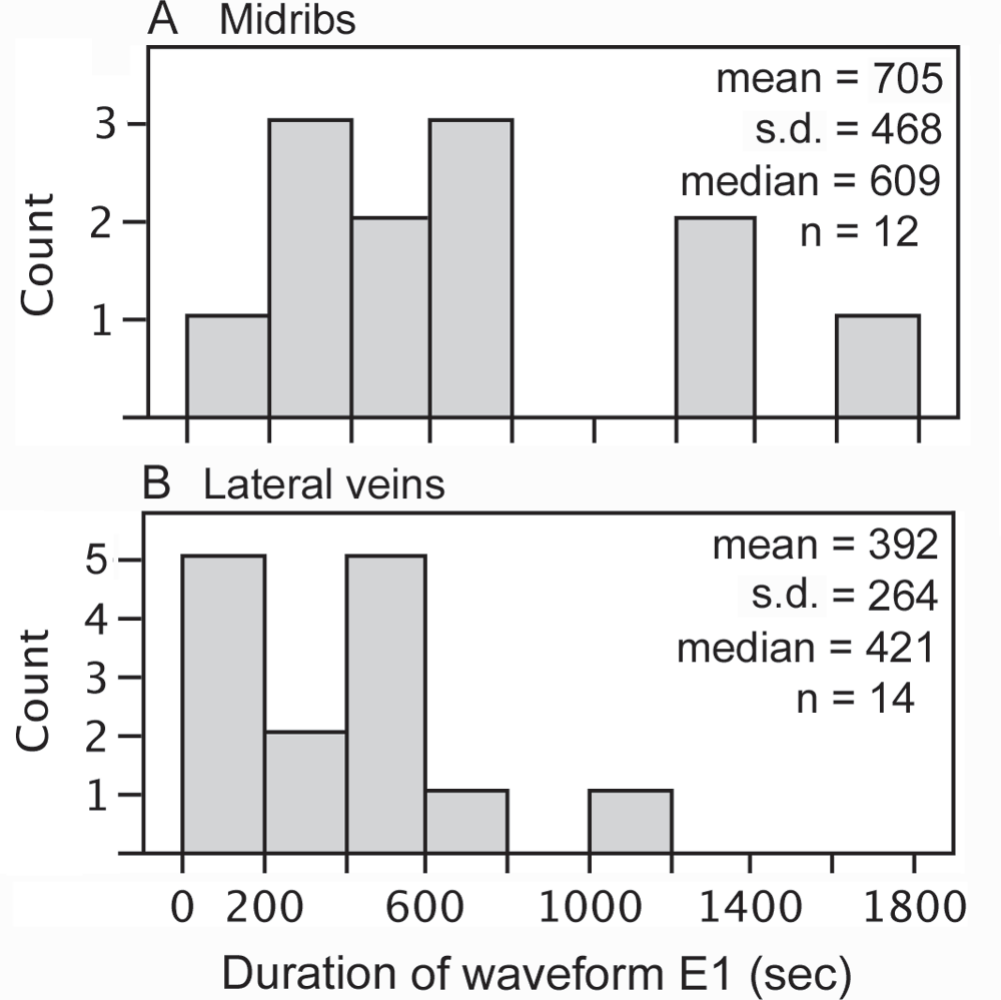
Frequency distributions of duration of sieve element salivation (E1) following application of a remote burn stimulus on midribs (A) and lateral veins (B). Duration of E1 was significantly greater on midribs than on lateral veins (*P*=0.0372, Wilcoxon test, normal approximation).

On both midribs and lateral veins, all aphids that were engaged in phloem ingestion (waveform E2) responded to a remote burn stimulus by switching from ingestion to salivation (waveform E1). E1 began 8.5±3.8 s (*n*=26) after application of the remote burn with no significant difference between midribs and lateral veins (*P*=0.0895, Wilcoxon test, normal approximation). The duration of E1 following burning was significantly longer on midribs than on lateral veins (705±468 versus 392±264 s; *P*=0.0372, Wilcoxon test, normal approximation; Fig. 2).

The proportion of aphids that immediately followed E1 by resuming phloem ingestion (E2) rather than going into pathway phase was marginally different between midribs (2 out of 12 aphids) and lateral veins (8 out of 14 aphids) (Fisher’s exact test *P*=0.0511). On lateral veins, the mean duration of E1 did not differ significantly between aphids that immediately followed E1 with E2 (449±135, *n*=8) and those that immediately followed E1 with pathway (316±379, *n*=6; *P*=0.1752, Wilcoxon test, normal approximation). However, the variance was much greater for those that followed E1 with pathway (*P*=0.0174, F-test). The number of aphids that immediately followed E1 with E2 on midribs was too low to make a meaningful comparison of E1 duration between aphids that followed E1 with E2 or pathway.

### Experiment 3: Effect of aphid salivation on dispersed forisome recovery

**Fig. 3.**
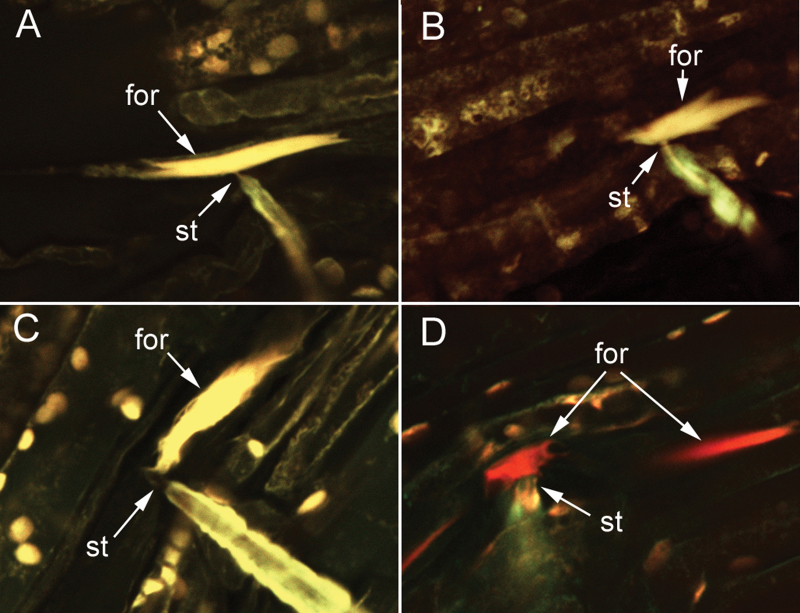
Confocal micrographs illustrating examples of forisomes (for) classified as (A) condensed; (B and C) intermediate; and (D) dispersed. Stylet tips (st) are in contact with the forisome in all 4 micrographs. Note in (D) that while the forisome in the penetrated sieve element is dispersed, a forisome in a neighbouring sieve element (right side of micrograph) is condensed. Samples were double stained with DiOC_7_(3) and sulphorhodamine 101 (Molecular Probes, Eugene, OR USA).

Only samples where forisomes could be traced with confidence to the penetrated sieve element were considered for this study; there were 29 such samples for aphids feeding on lateral veins and 28 for aphids feeding on midribs. Confocal micrographs showing examples of forisomes in dispersed, intermediate, and condensed states are presented in Figure 3.

**Fig. 4.**
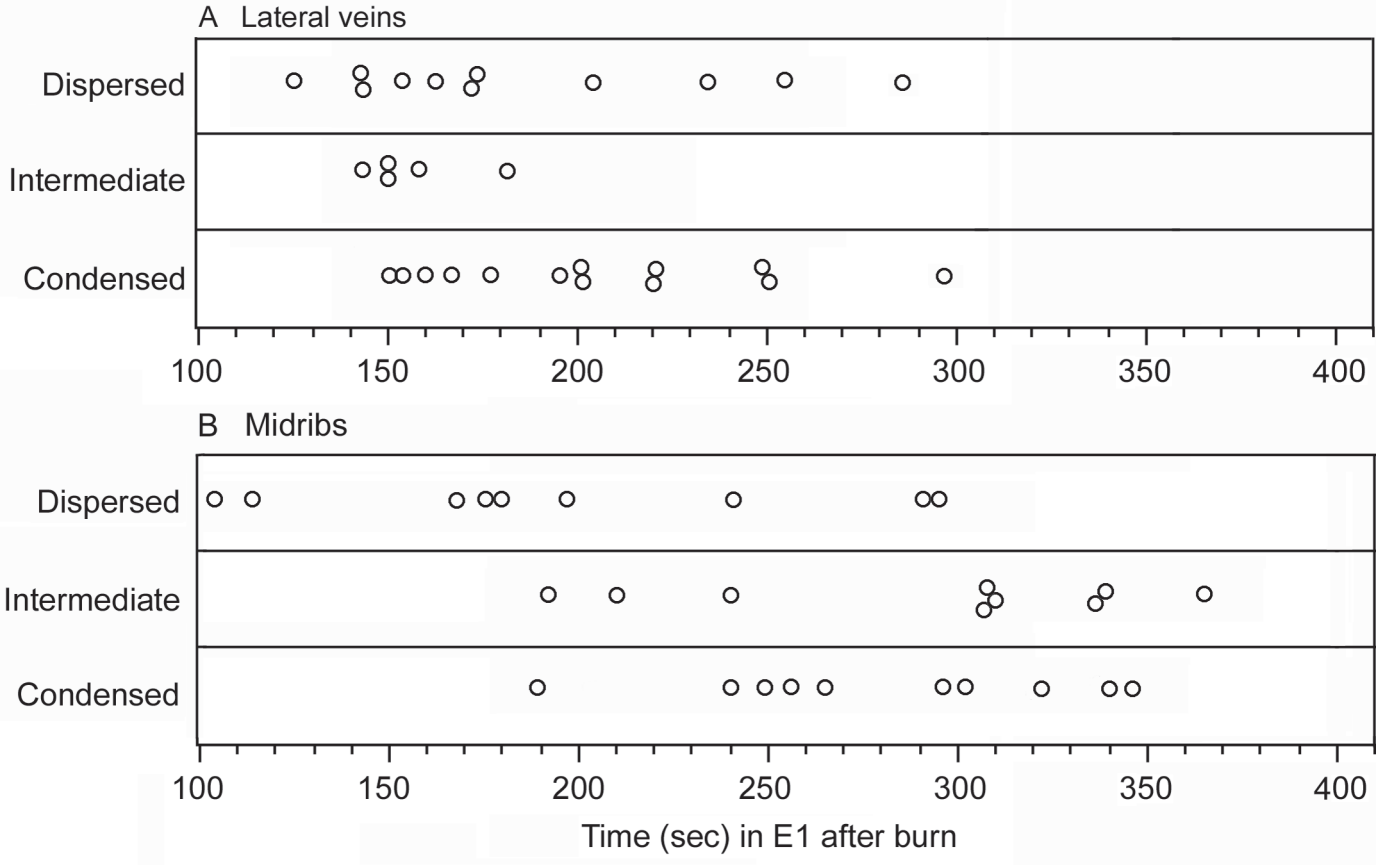
State of forisomes in sieve elements penetrated by pea aphid stylets after different durations of E1 salivation following application of a remote burn stimulus on lateral veins (A) and midribs (B). Each circle represents a different sample and is plotted according to the duration of time that the aphid had salivated into the sieve element prior to cryofixation. Data are plotted in three panels according to whether the forisome in the penetrated sieve element was condensed, dispersed, or in an intermediate state (Fig. 3).

Table 1 provides relevant data for each replicate: duration of remote burn-induced phloem salivation (E1) prior to cryofixation, the state of the forisome in the penetrated sieve element, and the state of forisomes in nearby sieve elements. Figure 4 plots the duration of E1 for each replicate in three panels according to whether the forisome in the penetrated sieve element was condensed, dispersed, or in an intermediate state.

**Table 1.** E1 duration and state of forisomes in the penetrated sieve element (SE) and nearby sieve elements in experiment 3

Midribs						Lateral veins					
Replicate	E1 duration (s)	State of forisome in penetrated SE	State of forisomes in nearby SEs (*n*, proportion)			Replicate	E1 duration (s)	State of forisome in penetrated SE	State of forisomes in nearby SEs (*n*, proportion)		
			Condensed	Intermediate	Dispersed				Condensed	Intermediate	Dispersed
1	104	Dispersed	3 (0.50)	0 (0.00)	3 (0.50)	1	125	Dispersed	0 (0.00)	0 (0.00)	8 (1.00)
2	114	Dispersed	1 (0.13)	0 (0.00)	7 (0.88)	2	143	Dispersed	1 (0.33)	1 (0.33)	1 (0.33)
3	168	Dispersed	1 (0.13)	0 (0.00)	7 (0.88)	3	143	Dispersed	1 (0.25)	1 (0.25)	2 (0.50)
4	176	Dispersed	0 (0.00)	1 (0.17)	5 (0.83)	4	143	Intermediate	4 (0.67)	0 (0.00)	2 (0.33)
5	180	Dispersed	1 (0.20)	0 (0.00)	4 (0.80)	5	150	Intermediate	2 (0.14)	1 (0.07)	11 (0.79)
6	189	Condensed	9 (0.82)	0 (0.00)	2 (0.18)	6	150	Condensed	1 (0.33)	0 (0.00)	2 (0.67)
7	192	Intermediate	3 (0.38)	2 (0.25)	3 (0.38)	7	150	Intermediate	2 (0.67)	0 (0.00)	1 (0.33)
8	197	Dispersed	6 (0.55)	1 (0.09)	4 (0.36)	8	153	Dispersed	3 (0.30)	1 (0.10)	6 (0.60)
9	210	Intermediate	5 (1.00)	0 (0.00)	0 (0.00)	9	154	Condensed	6 (1.00)	0 (0.00)	0 (0.00)
10	240	Intermediate	2 (0.67)	1 (0.33)	0 (0.00)	10	158	Intermediate	0 (0.00)	0 (0.00)	2 (1.00)
11	240	Condensed	8 (1.00)	0 (0.00)	0 (0.00)	11	160	Condensed	5 (1.00)	0 (0.00)	0 (0.00)
12	241	Dispersed	3 (0.75)	1 (0.25)	0 (0.00)	12	162	Dispersed	2 (0.67)	0 (0.00)	1 (0.33)
13	249	Condensed	3 (1.00)	0 (0.00)	0 (0.00)	13	167	Condensed	3 (1.00)	0 (0.00)	0 (0.00)
14	256	Condensed	5 (1.00)	0 (0.00)	0 (0.00)	14	172	Dispersed	7 (0.70)	1 (0.10)	2 (0.20)
15	265	Condensed	4 (0.57)	0 (0.00)	3 (0.43)	15	173	Dispersed	4 (0.57)	1 (0.14)	2 (0.29)
16	291	Dispersed	2 (0.50)	1 (0.25)	1 (0.25)	16	177	Condensed	5 (1.00)	0 (0.00)	0 (0.00)
17	295	Dispersed	4 (1.00)	0 (0.00)	0 (0.00)	17	182	Intermediate	2 (1.00)	0 (0.00)	0 (0.00)
18	296	Condensed	2 (1.00)	0 (0.00)	0 (0.00)	18	195	Condensed	7 (0.78)	0 (0.00)	2 (0.22)
19	302	Condensed	5 (0.83)	1 (0.17)	0 (0.00)	19	201	Condensed	8 (0.89)	0 (0.00)	1 (0.11)
20	307	Intermediate	6 (0.60)	4 (0.40)	0 (0.00)	20	201	Condensed	6 (1.00)	0 (0.00)	0 (0.00)
21	308	Intermediate	3 (1.00)	0 (0.00)	0 (0.00)	21	204	Dispersed	2 (0.33)	0 (0.00)	4 (0.67)
22	310	Intermediate	2 (0.67)	1 (0.33)	0 (0.00)	22	220	Condensed	1 (0.50)	0 (0.00)	1 (0.50)
23	322	Condensed	5 (1.00)	0 (0.00)	0 (0.00)	23	221	Condensed	2 (0.67)	1 (0.33)	0 (0.00)
24	337	Intermediate	4 (1.00)	0 (0.00)	0 (0.00)	24	234	Dispersed	5 (0.71)	0 (0.00)	2 (0.29)
25	339	Intermediate	6 (0.67)	3 (0.33)	0 (0.00)	25	248	Condensed	4 (0.67)	1 (0.17)	1 (0.17)
26	340	Condensed	2 (0.25)	5 (0.63)	1 (0.13)	26	250	Condensed	5 (1.00)	0 (0.00)	0 (0.00)
27	346	Condensed	6 (1.00)	0 (0.00)	0 (0.00)	27	254	Dispersed	2 (0.25)	6 (0.75)	0 (0.00)
28	365	Intermediate	4 (1.00)	0 (0.00)	0 (0.00)	28	286	Dispersed	7 (0.88)	0 (0.00)	1 (0.13)
						29	297	Condensed	11 (1.00)	0 (0.00)	0 (0.00)

To determine if E1 salivation accelerates the recovery of forisomes after a remote burn stimulus, the proportion of forisomes in each state (condensed, dispersed, or intermediate) was compared between sieve elements in which the aphid was salivating at the time of cryofixation and nearby non-penetrated sieve elements. Both methods of calculation yielded similar values (Table 2 and Supplementary Table S1, available at *JXB* online). The samples were divided into two groups according to E1 duration under the rationale that samples cryofixed after shorter E1 durations (i.e. shorter post-burn time) would be expected to have a greater proportion of dispersed forisomes in nearby non-penetrated sieve elements (the controls) than samples cryofixed after longer E1 durations (i.e. longer post-burn time). On midribs, the 28 samples were pooled into two groups: those cryofixed after the aphids engaged in E1 salivation for 104–197 s and those cryofixed after 210–365 s of E1 salivation (Table 2). This was based on a fairly distinct drop in the proportion of dispersed forisomes in nearby non-penetrated sieve elements between the 104–197 s group and the 210–365 s group (Table 1). On lateral veins, the 29 samples were pooled into two groups, those cryofixed after the aphids engaged in E1 salivation for 125–182 s and those cryofixed after 195–297 s of E1 salivation (Table 2). The proportion of forisomes in a dispersed state in nearby non-penetrated sieve elements for lateral veins was quite variable and did not exhibit as clean a drop over time as in the midribs (Table 1), so the division of the samples into two groups was based on a relatively large gap between the longest E1 in the first group (182 s) and the shortest E1 in the second group (195 s) (Table 1 and Fig. 4).

**Table 2.** State of forisomes (dispersed, intermediate, or condensed) in sieve elements (SEs) penetrated by the stylet tips (subjected to direct E1 salivation) and in nearby non-penetrated sieve elements (not subjected to direct E1 salivation)Values are % (*n*/total). Samples were pooled into two groups for lateral veins and midribs according to the duration of E1. In each sample, the forisome in the penetrated sieve element was scored as well as forisomes in several nearby non-penetrated sieve elements (as described in Materials and Methods). Consequently, the total number of forisomes are higher for non-penetrated sieve elements (where multiple forisomes per sample were scored) than for penetrated sieve elements (a single sieve element in each sample). An alternative way of calculating the proportion of forisomes in each state in non-penetrated sieve elements would be to calculate the proportions for each sample and then calculate the average proportion over all samples, so there is a single value per sample (see Materials and Methods). Proportions are calculated by that alternative method in Supplementary Table S1 and are very similar to those presented here: conclusions are unaffected.

State of forisome	Lateral veins				Midribs			
	E1=125–182 s		E1=195–297 s		E1=104–197 s		E1=210–365 s	
	Non-penetrated SEs	Penetrated SEs	Non-penetrated SEs	Penetrated SEs	Non-penetrated SEs	Penetrated SEs	Non-penetrated SEs	Penetrated SEs
Dispersed	43 (40/94)	41 (7/17)	15 (12/80)	33 (4/12)	56 (35/63)	75 (6/8)	5 (5/103)	15 (3/20)
Intermediate	6 (6/94)	29 (5/17)	10 (8/80)	0 (0/12)	6 (4/63)	13 (1/8)	17 (17/103)	40 (8/20)
Condensed	51 (48/94)	29 (5/17)	75 (60/80)	67 (8/12)	38 (24/63)	13 (1/8)	79 (81/103)	45 (9/20)

For lateral veins cryofixed after 125–182 s of E1 salivation, the proportions of forisomes in the dispersed state were similar between penetrated and non-penetrated sieve elements (41 and 43%; Table 2). Additionally, the proportion of forisomes in the condensed state was considerably lower in penetrated sieve elements compared to non-penetrated sieve elements (29 versus 51%; Table 2). Consequently, these data do not indicate a more rapid forisome recovery in the penetrated sieve elements.

For lateral veins cryofixed after 195–297 s of E1 salivation, the proportion of forisomes in the dispersed state in the penetrated sieve elements was over twice that of the non-penetrated sieve elements (33 versus 15%; Table 2). Again, these data argue against a more rapid forisome recovery in the penetrated sieve elements.

In penetrated sieve elements in midribs cryofixed after 104–197 s of E1 salivation, the proportion of forisomes in the dispersed state was higher (75 versus 56%; Table 2) and the proportion of forisomes in the condensed state was almost three times lower (13 versus 38%) than in non-penetrated sieve elements (Table 2). Thus once again, forisomes did not recover any faster in the penetrated sieve elements.

Finally, for penetrated sieve elements in midribs cryofixed after 210–365 s of E1 salivation, the proportion of forisomes in the dispersed state was higher (15 versus 5%; Table 2) and the proportion of forisomes in the condensed state lower (45 versus 79%; Table 2) than for non-penetrated sieve elements. As in the previous three comparisons, the data clearly show that forisomes subjected to direct E1 salivation do not recover faster than those in nearby sieve elements that were not subjected to direct E1 salivation.

## Discussion

Will *et al.* (2007) provided intriguing circumstantial evidence that aphid salivation can reverse sieve element occlusion, an ability that would be of enormous value to these obligate phloem sap feeders. They first used the remote burn technique while the vetch aphid, *M. viciae*, was feeding on midribs of faba bean leaves and observed that about 16 s after burning, 10 out of 12 aphids switched from phloem sap ingestion to salivation into the sieve element and then eventually resumed their normal phloem sap ingestion behaviour about 8.3min later. Next, they collected vetch aphid saliva from artificial diet that was fed upon by thousands of aphids, concentrated the diet (diet fed upon by 6750 aphids was concentrated to 4 μl), and found that the concentrate caused forisomes to transform from a dispersed state to a condensed state in an *in vitro* assay. Finally, they identified salivary proteins with calcium-binding properties, which is relevant because calcium chelators, such as EDTA, can also transform forisomes from a dispersed state to a condensed state *in vivo* (Knoblauch *et al.*, 2001).

Will *et al.* (2007) concluded that resumption of normal phloem sap ingestion behaviour after 8.3min of salivation occurred because salivation triggered the forisomes to revert from a dispersed state to a condensed state, allowing the resumption of sap flow. However, forisomes revert back to a condensed state on their own about 7–15min after a remote burn stimulus (Furch *et al.*, 2007) (however, as will be discussed, forisomes appear to revert even faster than 7–15min). Will *et al.* (2007) did not examine the condition of the forisomes, so it remains uncertain if the aphid salivation in that study actually reversed forisome dispersal. The ability of saliva concentrated from diet fed upon by aphids provided tantalizing evidence that aphid saliva is capable of reversing forisome dispersal; however, it is far from certain that the results of an *in vitro* assay using concentrated diet fed upon by so many aphids is equivalent to the natural situation of a single aphid salivating into a living sieve element. Consequently, to address these uncertainties, the recently developed methodology of Walker and Medina-Ortega (2012) was used to directly observe the state of forisomes penetrated by aphid stylets following remote burn-induced salivation.

Data from the present *in vivo* study provided no evidence that pea aphid salivation reverses forisome dispersal. In sieve elements being fed upon by aphids, the forisomes were almost always (98% of the time) close to or in contact with the stylet tips (Fig. 3), presumably drawn there by the flow of sap into the aphid’s food canal (forisomes tend to be carried along with sap flow in the sieve element; Peters *et al.* 2006). Consequently, E1 saliva was secreted directly onto the forisome; yet the data in Table 2 indicate that dispersed forisomes did not revert back to a condensed state any sooner in the penetrated sieve element than in nearby non-penetrated sieve elements.

Interestingly, the trends in the data (Table 2 and Supplementary Table S1) would be more supportive of the hypothesis that forisome recovery in penetrated sieve elements was somewhat slower rather than faster than recovery in non-penetrated sieve elements. As a possible mechanism for that alternative hypothesis, penetration of the plasmalemma and sieve element reticulum by the stylet tips may compromise the functioning of the calcium pumps that remove Ca^2+^ from the sieve element lumen. Forisomes disperse in response to a remote burn stimulus due to an influx of Ca^2+^ into the sieve element from the apoplast and from the parietal sieve element reticulum (Furch *et al.*, 2007, 2009; Hafke *et al.*, 2009) and the subsequent recovery to the condensed state is presumably due to removal of Ca^2+^ from the sieve element lumen and back to these storage compartments. Compromising the pumps responsible for Ca^2+^ removal would result in slower recovery of forisomes. Another possible mechanism for slower forisome recovery in penetrated sieve elements is that calcium channels in sieve elements are concentrated near the sieve plates where forisomes are usually located (Furch *et al.*, 2009; Hafke *et al.*, 2009), but in penetrated sieve elements, forisomes are almost always in close contact with the stylet tips (Fig. 3) which do not necessarily penetrate near the sieve plates. A lower density of Ca^2+^ channels away from the sieve plates may slow down both the initial dispersion and subsequent recovery of forisomes.

Forisomes recovered faster in lateral veins compared to midribs (Fig. 1). The faster recovery in lateral veins may be due to the smaller size of sieve elements in lateral veins that would provide a greater surface area to volume ratio, which would facilitate removal of Ca^2+^ from the lumen. Alternatively, stimulus strength is reported to affect forisome reactivity (Hafke *et al.*, 2009); therefore, the two methods of applying the remote burn stimulus (open flame at the apex of the midrib versus a soldering iron at the apex of the lateral veins) may have affected the intensity of calcium influx, and consequently affected the time required for Ca^2+^ concentration to be reduced below the dispersion threshold.

Forisome recovery time after remote burning varied considerably among replicates (Fig. 1 and Supplementary Fig. S1) which is consistent with wide variation reported in previous studies (e.g. 7–15min in Furch *et al.*, 2007); however, forisome recovery time was considerably faster in this study (Fig. 1) compared to previous reports. One potential reason for this discrepancy is that previous studies used a faba bean cultivar (cv. Witkiem major) different than the present study. However, repeating experiments 1 and 2 on cv. Witkiem major indicated that forisome recovery time following a remote burn stimulus was similar for cv. Witkiem major (Supplementary Fig. S1) and cv. Windsor (Fig. 1). Other potential differences between this study and previous studies such as degree of burning, plant growing conditions, and ambient room conditions may also have affected forisome recovery time, but the difference in methodology between Furch *et al.* (2007, 2009) and the present study may be the most likely reason for the faster forisome recovery in the present study. Furch *et al.* (2007, 2009) measured forisome recovery time while observing living sieve elements with confocal microscopy. To do this, they exposed the phloem by excising overlaying cortical cells and inundated the exposed area with a bathing solution that contained calcium. Forisome recovery may have been slowed down by the combined effects of excision damage and elevated apoplastic calcium caused by the bathing solution (Knoblauch *et al.*, 2001). In the present study, the phloem was undisturbed between application of the remote burn stimulus and cryofixation. This would seem likely to provide a more artefact-free measurement of forisome recovery time.

Will *et al.* (2007) also used cv. Witkiem major, but the consequence of the difference in cultivar between their study and the present study is probably negligible. The forisome response of cv. Witkiem major to remote burning was similar to that of cv. Windsor (compare Fig. 1 with Supplementary Fig. S1), and the aphid salivation response also was similar (compare Fig. 2 with Supplementary Fig. S2). There were some other differences in addition to the cultivar between the present study and Will *et al.* (2007). Will *et al.* (2007) used the vetch aphid, *M. viciae*, which does not occur in North America; consequently, due to agricultural regulations, the present study was unable to use this aphid and instead used the pea aphid, *A. pisum*. EPGs were recorded from aphids feeding on midribs 6cm from the vein apex in Will *et al.* (2007). In the present study, the distance was 3cm, the distance used by Furch *et al.* (2007) to determine the forisome response to leaf tip burning. In Will *et al.* (2007), 10 out of 12 aphids feeding on midribs went back to phloem sap ingestion (waveform E2) after remote burn-induced salivation; whereas in the present study on midribs, only two out of 12 aphids did so, and the rest went to pathway. On lateral veins in the present study, the proportion was higher: eight out of 14 aphids went back to phloem sap ingestion after remote burn-induced salivation (Will *et al.* 2007 did not use lateral veins).

While the present results indicate that pea aphid E1 salivation does not reverse remote burn-induced forisome dispersal any faster than the forisomes would recover on their own, the results do not rule out a potential role of E1 salivation in interfering with sieve element occlusion. Forisome reaction is quantitatively related to the strength of the stimulus, and leaf burning, a very unnatural stimulus, is the strongest stimulus reported so far (Hafke *et al.*, 2009). It is possible that E1 salivation would be more effective at reversing forisome dispersal triggered by weaker, more natural stimuli, although if it has any ability to reverse forisome dispersal, one would expect to see at least a slightly faster recovery time in penetrated sieve elements even if the initial dispersal stimulus was very strong. The results of this study do not show that. Another possibility is that E1 salivation may be more important in preventing rather than reversing forisome dispersal. Initial penetration of the sieve element may cause some leakage of Ca^2+^ into the sieve element at the penetration site or might cause a slight loss of turgor that allows a slow influx of Ca^2+^ through mechano-sensitive Ca^2+^ channels; thus the bout of E1 salivation that always occurs for the first 30–60 s of phloem phase immediately after initial penetration of the sieve element may function to sequester this Ca^2+^ and prevent it from reaching a threshold that would trigger sieve element occlusion. It also is possible that E1 salivation is primarily targeting callose synthesis rather than SEO protein plugs (forisome dispersal). Callose plugs last longer than SEO protein plugs (Furch *et al.*, 2007) and thus would be more problematic for aphids, and callose synthesis may have a much higher calcium threshold than forisome dispersal (Furch *et al.*, 2007), so it presumably would be easier for aphids to suppress.

Switching behaviour from phloem sap ingestion to salivation into the sieve element in response to a remote burn stimulus seems to be a universal response among aphid species (Will *et al.*, 2009). This gives rise to the question: what triggers the switch to E1? In addressing this question, the present authors found that, in response to a remote burn, the switch from E2 to E1 occurs before the forisome in the penetrated sieve element disperses (K.J. Medina-Ortega and G.P. Walker, unpublished data); therefore the switch does not appear to be triggered by dispersal of the forisome in the sieve element upon which they are feeding. Furthermore, the maxillary stylets are not innervated (Forbes, 1969, 1977), so there is no known sensory apparatus that would seem capable of detecting a change in the physical state of the forisome. Instead, the switch may be triggered by a sudden drop in sieve element turgor pressure. In response to a remote burn, sieve elements upstream of the aphid feeding site occlude before the wave of occlusion reaches the sieve element on which the aphid is feeding. Upstream occlusion results in a downstream decrease in turgor pressure (Gould *et al.*, 2004); consequently, it is quite feasible that in response to remote burning, the aphids experience a sudden turgor drop before the forisome in their sieve element disperses. This could explain why the switch to E1 occurs before dispersal of the forisome in the penetrated sieve element. In support of this hypothesis, Will *et al.* (2008) demonstrated that the aphid *Myzus persicae* (Sulzer) switched from ingestion to salivation when the turgor pressure in an artificial feeding system was suddenly reduced.

The duration of E1 salivation following burning is considerably longer than the time it takes for forisomes to revert to a condensed state (i.e. compare Figs. 1 and 2). Additionally, all samples in experiment 3 were cryofixed while the aphids were still in E1 salivation behaviour, yet in many of these samples, the forisome in the sieve element into which they were salivating had already reverted back to the condensed state (Fig. 4). Taken together, this indicates that recovery of the forisome is not the cue that triggers the aphids to terminate E1. If the original switch to E1 was triggered by a drop in turgor pressure as suggested previously, then the aphid may be waiting for the turgor pressure to recover before terminating E1 and resuming E2. In response to a remote burn stimulus, callose deposition lasts much longer than forisome dispersal (Furch *et al.*, 2007), so it seems reasonable to hypothesize that reduced turgor would last longer than forisome dispersal.

Finally comes the question: if E1 salivation is incapable of reversing forisome dispersal, then what is the purpose of switching from E2 to E1 following a remote burn stimulus? The switch to E1 could be an attempt to restore sieve element turgor, but it is doubtful if aphids would be capable of injecting enough saliva to affect the turgor. Perhaps aphids perceive reduced turgor as an indication of callose building up at the sieve plates and, as proposed previously, the target of the salivation may be inhibiting callose synthase by reducing Ca^2+^. While aphids are capable of ingesting fluids at zero or negative pressure (from artificial diets or xylem, respectively), it likely would be worth the investment of saliva to maintain a high turgor and thus a high rate of ingestion. Lastly, it should be kept in mind that remote burning is a very strong and unnatural trigger of sieve element occlusion (Hafke *et al.*, 2009). As suggested previously, the direct cue that triggers aphids to switch to E1 may be a drop in turgor, and E1 salivation may function primarily to prevent sieve element occlusion by sequestering Ca^2+^ leaking through turgor-activated mechano-sensitive Ca^2+^ channels. Aphid saliva may be effective at sequestering low-level Ca^2+^ influx caused by natural fluctuations in turgor but may be overwhelmed by a large influx of Ca^2+^ triggered by remote burning.

In conclusion, it is obvious that many questions remain regarding the interaction between aphids and phloem occlusion mechanisms. As phloem occlusion is a potential means of plant resistance against this important group of agricultural pests, answers to these questions would be of more than just academic interest.

## Supplementary material

Supplementary data are available at *JXB* online.

Supplementary Fig. S1. Forisome recovery time after a remote burn stimulus for *V. faba* cv. Witkiem major.

Supplementary Fig. S2. Frequency distributions of duration of sieve element salivation (E1) following application of a remote burn stimulus on midribs and lateral veins for *V. faba* cv. Witkiem major.

Supplementary Table S1. State of forisomes (dispersed, intermediate, or condensed) in sieve elements penetrated by the stylet tips and in nearby non-penetrated sieve elements. Same as Table 2 except the proportion of forisomes in each state in non-penetrated sieve elements is calculated differently.

Supplementary Data
