# Supplementary material for: Does aphid salivation affect phloem sieve element occlusion in vivo?
Source: J Exp Bot. 2013 Oct 14;64(18):5525–35. doi: 10.1093/jxb/ert325 (PMC3871815; doi:10.1093/jxb/ert325)
Supplement: Supplementary Data [file supp_64_18_5525__index.html]

Does aphid salivation affect phloem sieve element occlusion in vivo? — Does aphid salivation affect phloem sieve element occlusion in vivo? — Supplementary Data 

# Does aphid salivation affect phloem sieve element occlusion *in vivo*?

## Supplementary Data

Data files

**Files in this Data Supplement:**

- Supplementary Data - Supplementary Data
