# Supplementary material for: Does aphid salivation affect phloem sieve element occlusion in vivo?
Source: J Exp Bot. 2013 Oct 14;64(18):5525–35. doi: 10.1093/jxb/ert325 (PMC3871815; doi:10.1093/jxb/ert325)
Supplement: Supplementary Data [file supp_ert325_jexbot103325_file001.pdf]

Table S1. Same as Table 1 except the proportion of forisomes in each state in non-penetrated sieve elements (SEs) is calculated differently (see Materials and Methods). In Table 1, first the total number of forisomes in each state over all the samples was summed and then proportions were calculated based on these sums. In this Table (S1), the proportions of forisomes in each state were calculated for each sample and then the average over all samples was calculated. Conclusions are the same regardless of the method of calculation.

	Lateral Veins E1 = 125 – 182 sec		Lateral Veins E1 = 195 – 297 sec		Midribs E1 = 104 – 197 sec		Midribs E1 = 210 – 365 sec	
State of forisome	Non-penetrated SEs	Penetrated SEs	Non-penetrated SEs	Penetrated SEs	Non-penetrated SEs	Penetrated SEs	Non-penetrated SEs	Penetrated SEs
dispersed	37%	41% (7/17)	17%	33% (4/12)	60%	75% (6/8)	4%	15% (3/20)
intermediate	6%	29% (5/17)	10%	0% (0/12)	6%	13% (1/8)	13%	40% (8/20)
condensed	57%	29% (5/17)	72%	67% (8/12)	34%	13% (1/8)	83%	45% (9/20)

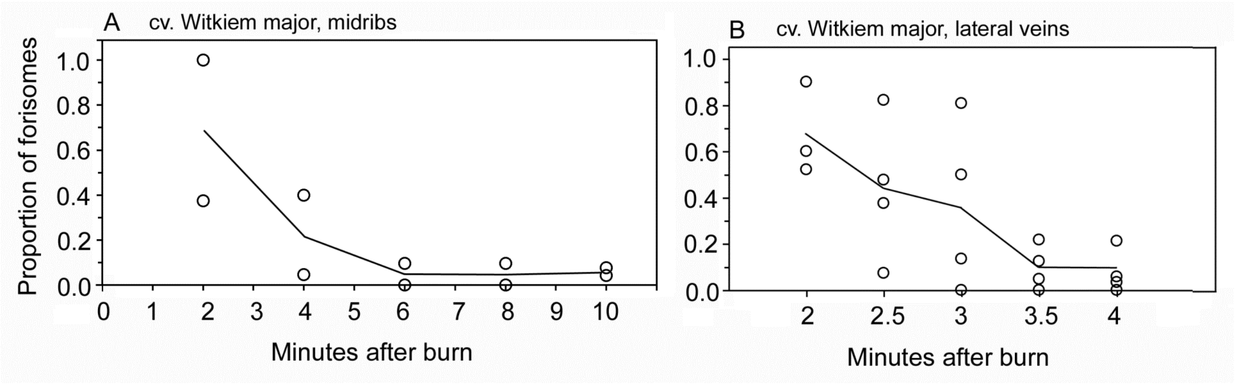

Fig. S1. Proportion of forisomes in a dispersed state in midribs (A) and lateral veins (B) at different times after application of a remote burn stimulus for cv. Witkiem major. Circles plot data from individual replicates (n= 12-40 forisomes examined in each replicate); lines plot average of all replicates at each time. In controls (no remote burn), proportion of forisomes in a dispersed state was zero in all replicates for both vein types (n = 2 replicates for midribs and n = 3 replicates for lateral veins; data not plotted).

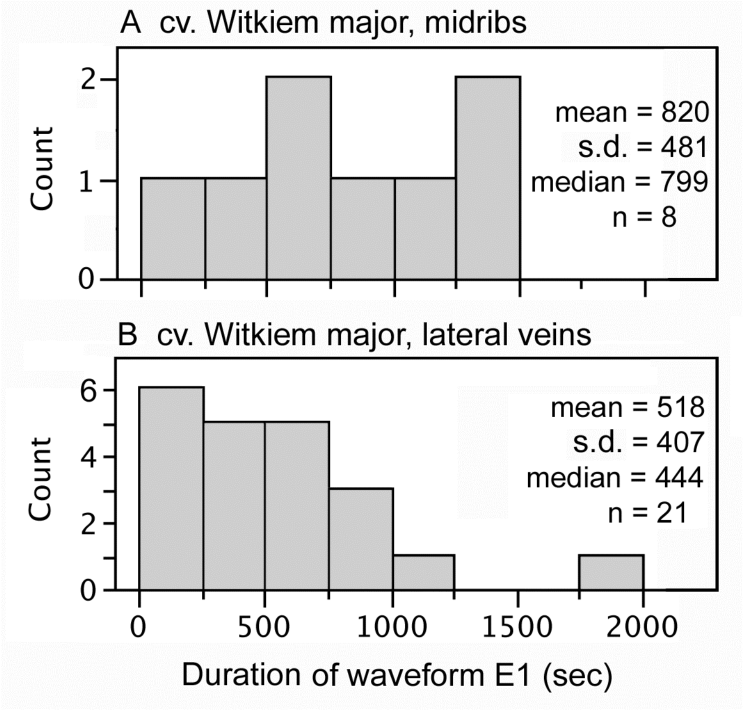

Fig. S2. Frequency distributions of duration of sieve element salivation (E1) following application of a remote burn stimulus on midribs (A) and lateral veins (B). Duration of E1 was not significantly different between midribs and lateral veins ($P = 0.1021$, Wilcoxon test, normal approximation).
